# Multi-Scale Attention Fusion Gesture-Recognition Algorithm Based on Strain Sensors

**DOI:** 10.3390/s25134200

**Published:** 2025-07-05

**Authors:** Zhiqiang Zhang, Jun Cai, Xueyu Dai, Hui Xiao

**Affiliations:** School of Electrical and Information Engineering, Anhui University of Science and Technology, Huainan 232001, China; zhang1021415@163.com (Z.Z.); m19965925649@163.com (X.D.); hui157562@163.com (H.X.)

**Keywords:** dynamic-gesture recognition, strain sensors, hybrid attention mechanism, multi-scale feature fusion, cross-modal recognition

## Abstract

Surface electromyography (sEMG) signals are commonly employed for dynamic-gesture recognition. However, their robustness is often compromised by individual variability and sensor placement inconsistencies, limiting their reliability in complex and unconstrained scenarios. In contrast, strain-gauge signals offer enhanced environmental adaptability by stably capturing joint deformation processes. To address the challenges posed by the multi-channel, temporal, and amplitude-varying nature of strain signals, this paper proposes a lightweight hybrid attention network, termed MACLiteNet. The network integrates a local temporal modeling branch, a multi-scale fusion module, and a channel reconstruction mechanism to jointly capture local dynamic transitions and inter-channel structural correlations. Experimental evaluations conducted on both a self-collected strain-gauge dataset and the public sEMG benchmark NinaPro DB1 demonstrate that MACLiteNet achieves recognition accuracies of 99.71% and 98.45%, respectively, with only 0.22M parameters and a computational cost as low as 0.10 GFLOPs. Extensive experimental results demonstrate that the proposed method achieves superior performance in terms of accuracy, efficiency, and cross-modal generalization, offering a promising solution for building efficient and reliable strain-driven interactive systems.

## 1. Introduction

As an intuitive and expressive interaction modality, gestures involve coordinated movements of finger joints, enabling the execution of complex and delicate actions while effectively conveying rich semantic information. Accurate gesture recognition plays a pivotal role in a wide range of domains, including medical assistance [1], robotic control [2], augmented reality gaming [3], and HCI systems [4,5].

Given the high flexibility of the fingers in fine motor control, gesture-recognition approaches based on bioelectrical and biomechanical signals have gained increasing attention in recent years. These methods offer several advantages, such as strong resistance to environmental interference, independence from external imaging devices, and suitability for wearable deployment. Gesture recognition is typically categorized into static and dynamic gestures. “Static gestures” refers to fixed hand postures without temporal variations [6], while dynamic gestures involve a sequence of continuous movements within a short duration. The temporal dynamics embedded in dynamic gestures increase the complexity of the recognition task, yet offer higher practical value.

The sEMG, as a representative bioelectrical signal, effectively captures the electrophysiological activity of muscles during movement and has been widely adopted in dynamic-gesture recognition tasks [7]. However, sEMG signals face inherent limitations: they are less effective in detecting static gestures, highly sensitive to electrode placement and skin conditions, and insufficient for the capture of deep muscle activity. These factors hinder the generalization and robustness of sEMG-based methods in cross-user and cross-environment scenarios.

In contrast, strain sensors—characterized by their high sensitivity, flexibility, and low cost—can be conformally attached to finger joints to directly capture strain signals caused by joint bending in real-time. These signals exhibit clear temporal structure and action-specific patterns, providing stable and efficient angular information for dynamic-gesture recognition. Nevertheless, the extraction of informative multi-scale and temporal structural features from strain signals to effectively represent motion characteristics remains a critical challenge.

To address these challenges, this paper proposes a multi-scale feature modeling network for gesture recognition. In addition, to evaluate the generalization capability of the model, we conduct cross-modal comparative experiments using a public sEMG dataset. The results demonstrate that the proposed model achieves superior performance in both strain and sEMG modalities, highlighting its strong transferability and practical potential.

The main contributions of this study are summarized as follows:

1. A standardized multi-channel strain signal acquisition system and gesture execution protocol are developed, tailored for dynamic-gesture analysis.
2. A lightweight gesture-recognition network, MACLiteNet, is designed, incorporating causal temporal modeling and attention mechanisms.
3. A multi-scale feature enhancement module and a channel reconstruction mechanism are introduced to improve the model’s discriminative capability for complex gestures.
4. The proposed method demonstrates strong performance on both strain-based and public sEMG datasets, validating its transferability and architectural generality in multimodal dynamic-signal recognition tasks.

## 2. Related Work

### 2.1. Sensor Technologies and Data Acquisition Methods

In the field of dynamic-gesture recognition, the choice of sensing modality and the data acquisition strategy fundamentally determine the system’s overall performance and adaptability. The sEMG, a commonly used bioelectrical signal, reflects the electrophysiological activity generated by muscle contractions and has been widely applied in gesture recognition, neural prosthetics, and human–machine interaction scenarios. Researchers often transform sEMG signals into image-like representations or sequential signal streams, leveraging deep learning models such as CNNs to extract latent electromyographic patterns and action-related semantics [8,9].

However, sEMG-based methods suffer from significant inter-subject variability, which hinders their robustness in cross-user applications. Their sensitivity to electrode placement, skin condition, and muscle fatigue further limits their generalization capability in real-world settings.

In contrast to sEMG signals, strain signals exhibit clearer temporal structures and stronger correlations with joint movement dynamics. Strain sensors measure the subtle mechanical deformations caused by joint bending through their continuous resistance changes, offering advantages such as flexible attachment, low cost, and low power consumption [10]. In recent years, strain sensors have gradually become a promising direction for dynamic-gesture recognition. In particular, advances in sensor materials and fabrication techniques have further expanded the application potential of strain sensors in human–machine interaction. For example, Nikitina et al. [11] proposed a laser-processed strain sensor based on multi-walled carbon nanotube (MWCNT) networks embedded in a silicone elastomer. The sensor demonstrated excellent electrical conductivity, a high gauge factor, and low hysteresis, validating the feasibility of material-driven performance improvements in wearable strain sensing. Building upon these developments, an increasing number of research efforts have explored the direct attachment of strain sensors to finger joints to capture real-time angular variations. This approach enables the construction of multi-channel, temporally continuous motion-representation matrices, providing a stable and reliable signal foundation for dynamic-gesture recognition. Nevertheless, like other mechanical sensing devices, strain sensors exhibit inherent limitations, particularly hysteresis effects and sensitivity to precise placement, which may affect signal stability and consistency.

### 2.2. Gesture Recognition Based on Traditional Machine Learning and Deep Learning

Early gesture-recognition methods were predominantly based on traditional machine learning models, which typically involved manually extracting time-domain, frequency-domain, or wavelet-domain features from raw sensor data and feeding them into classifiers such as SVM [12], LDA [13], decision trees [14], HMM [15], and GMM [16]. These approaches were often sensitive to small datasets and heavily reliant on handcrafted features, making them less effective in handling high-dimensional, multi-channel, and dynamically changing gesture data.

In recent years, deep learning methods have rapidly advanced gesture recognition due to their end-to-end learning capabilities. CNNs, in particular, have demonstrated strong performance in extracting local features and modeling spatial structures, leading to their wide adoption in multi-channel physiological signal analysis. Luo et al. [17] developed a ResNet50-based sEMG image recognition model that effectively captured local texture features of actions while maintaining low computational complexity, achieving accuracies of 87.94% and 87.04% on the NinaPro DB1 and DB5 datasets, respectively, validating the feasibility of CNNs in physiological signal modeling.

To enhance local perception and channel selectivity, Shen et al. [18] proposed a lightweight channel attention CNN (ICA-CNN), incorporating an improved attention mechanism to optimize the response to important channels while maintaining low inference latency. Ahmed et al. [19] introduced Tamura texture features and a generic additive module into the ResNet50 backbone, constructing the Tamura–ResNet50–GAM model, which improved fine-grained texture discrimination in gesture images and achieved 96% accuracy on the American Sign Language dataset.

Despite these advancements, convolutional models still struggle with capturing long-range dependencies. Traditional recurrent neural networks (RNNs) are limited by sequential computation and vanishing gradients, making them less effective in modeling long-term temporal relationships.

Transformer architectures have emerged as a powerful alternative for temporal sequence modeling due to their global self-attention mechanism. Dosovitskiy et al. [20] introduced the Vision Transformer (ViT), which partitions images into fixed-size patches, encodes them as tokens, and processes them through a standard Transformer encoder, enabling global modeling of spatial structures and achieving performance comparable to, or better than, CNNs on large-scale datasets.

Subsequently, Liu et al. [21] proposed the Swin Transformer, which restricts attention to local windows and shifts window positions to enhance inter-window interactions, supporting hierarchical feature extraction and making the process suitable for high-resolution image modeling in complex scenarios. Mehta et al. [22] further introduced MobileViT, a lightweight structure designed for mobile platforms that combines local convolution with global self-attention to achieve efficient spatiotemporal modeling under low-latency conditions.

### 2.3. Hybrid Architectures and Multi-Branch Fusion Strategies

As gesture-recognition tasks demand greater depth in modeling, temporal sensitivity, and multimodal fusion capability, single-path architectures have become inadequate for capturing complex dynamic patterns. To address this, researchers have proposed various hybrid and multi-branch architectures that integrate local convolution modules, temporal modeling components, and multimodal pathways to capture dynamic information at different scales and semantic levels.

#### 2.3.1. CNN–Temporal Hybrid Structures

To compensate for the temporal modeling limitations of CNNs, some studies introduce RNNs in a serial or parallel fashion to strengthen the model’s capability for sequence learning.

Li et al. [23] proposed the MS-CLSTM model, which integrates multi-scale convolutional branches with bidirectional LSTM modules on top of a CNN backbone. By incorporating residual attention mechanisms, the model enhances channel perception and efficiently fuses spatial–temporal features, achieving 89.50% and 84.88% accuracies on NinaPro DB2 and DB4, respectively, outperforming traditional single-branch networks.

Rajalakshmi et al. [24] presented a hybrid CNN–LSTM architecture in which CNNs extract spatial features and LSTMs capture long-term temporal dependencies. Their results demonstrate that the local–global collaborative design significantly enhances dynamic-gesture modeling.

#### 2.3.2. Multi-Stream Feature Fusion Strategies

Multi-branch fusion structures extract diverse features in parallel channels, enhancing the model’s ability to perceive modality synergy, channel variability, and temporal alignment.

Peng et al. [25] proposed MSFF-Net, a multi-stream convolutional network that independently extracts features from each input channel. It incorporates residual attention and temporal alignment mechanisms at various stages to form an early–late fusion pathway. This method achieved 89.52% accuracy on the NinaPro DB2 dataset, validating its effectiveness in cross-channel modeling and temporal alignment.

Balaji et al. [26] introduced MFHAN, a model employing hierarchical self-attention encoders to represent image patches and cross-modal attention in order to align heterogeneous inputs such as images and skeleton data. Its extended version, MFHAN-2S, achieved 93.21% accuracy on the SHREC’17 dataset, significantly outperforming single-modal attention models.

#### 2.3.3. Trends in Module Fusion and Modeling Directions

Recent hybrid and multi-branch designs primarily focus on the following three directions:

1. Spatio-temporal decoupled modeling: Combining CNNs with LSTM or Transformer modules to separately model spatial features and temporal dynamics.
2. Multi-branch channel fusion: Constructing multi-stream architectures to aggregate features from different modalities or channels, enhancing the representation of heterogeneous inputs.
3. Attention-enhanced coordination: Incorporating residual, channel, or cross-modal attention mechanisms to improve the model’s focus on key regions and its fusion discrimination ability.

### 2.4. Motivation

Inspired by the above research, this study is motivated by the following observations:

1. Compared to sEMG signals, strain sensors offer greater stability and robustness against interference. While sEMG signals often suffer from ambiguity during static postures or deep muscle activity, strain sensors can continuously detect joint deformation, providing more reliable amplitude information for dynamic-gesture recognition.
2. The current mainstream gesture-recognition models, despite demonstrating good performance on standard tasks, still face accuracy bottlenecks in complex scenarios with subtle channel responses, rapid motion rhythms, or similar gesture postures. This is primarily due to limited dynamic feature extraction and insufficient modeling of inter-channel structural relationships. Therefore, designing a model with stronger discriminative power and global perceptive capacity is crucial.
3. Strain signals, when captured across multiple channels, contain rich inter-channel dependencies and spatial–temporal features. Relying on a single type of network structure is inadequate to model such composite relationships effectively. Especially in cases of variable gesture durations or complex posture transitions, a model lacking both short-term sensitivity and long-range dependency modeling may fail to generate stable and discriminative representations.

These challenges indicate that an effective dynamic-gesture recognition model should possess both an efficient short-term feature-extraction capability and a strong global modeling ability, while maintaining structural compactness for real-world applicability. However, conventional sequence-modeling approaches face inherent trade-offs in this regard. Recurrent neural networks such as LSTM and BiLSTM, though widely used, rely on sequential computations, resulting in increased inference latency and limited parallelizability. Pure Transformer-based models, while capable of global dependency modeling, typically introduce higher model complexity and computational demands. Hybrid architectures such as CNN-LSTM combine convolutional and recurrent modules and can achieve good performance, but often require larger model sizes to compensate for their limited global modeling capability.

To address these limitations, this study proposes MACLiteNet, a lightweight hybrid model designed to meet the challenges of dynamic-gesture recognition, especially in complex, multi-channel strain signal applications. Compared to conventional hybrid approaches, MACLiteNet achieves a more favorable balance between recognition accuracy, model compactness, and computational efficiency, making it well-suited for real-time applications.

## 3. Methodology

### 3.1. Strain Sensor Signal Acquisition System

**Figure 1:**
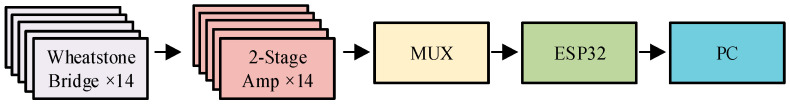
Structural diagram of the multi-channel strain signal acquisition and processing system.

As shown in Figure 1, this paper constructs a multi-channel dynamic strain signal acquisition and processing framework based on an ESP32 chip (Espressif Systems, Shanghai, China). The system consists of multiple functional modules, including a 14-channel strain signal acquisition module constructed using a Wheatstone bridge and two-stage amplification circuit, an analog multiplexer (MUX) multiplexing module for signal switching, and a parallel redundant sampling module composed of 5 ADC channels built into the ESP32 chip. In addition, the system integrates ESP32’s native Wi-Fi communication function to achieve real-time wireless transmission of sampled data.

To improve signal quality and system robustness, a lightweight signal-filtering mechanism is embedded to suppress instantaneous spikes and high-frequency noise during sampling. The specific processing steps include the following three tasks:

1. Outlier elimination: Discard the maximum and minimum values among the five ADC samples to remove transient interference;
2. Mean fusion: Average the remaining three values to obtain a more stable output voltage;
3. First-order low-pass filtering: Smooth the signal variation, preserve motion trend features, and enhance discriminability for subsequent recognition.

The system operates at a fixed sampling frequency of 100 Hz, at which the MUX cyclically traverses the 14 strain channels once per second to collect a complete data frame. To reduce baseline shifts caused by environmental and individual differences, the system sets an initial reference voltage of 2.5 V under a static, non-deformed condition, serving as the baseline for subsequent signal normalization.

Table 1 summarizes the main parameter configurations of the system, including sampling frequency, number of channels, processor architecture, and circuit dimensions, demonstrating its integrated optimization in volume, performance, and communication capabilities.

In this study, 14 strain-gauge sensors are deployed on the major flexion and extension joints of the five fingers to capture the surface strain responses associated with joint movements. Specifically, the thumb is equipped with one sensor on the metacarpophalangeal (MCP) joint and one on the interphalangeal (IP) joint. For each of the remaining four fingers (index, middle, ring, and little finger), one sensor is placed on each of the following joints: MCP, proximal interphalangeal (PIP), and distal interphalangeal (DIP), resulting in a total of 14 sensors. All sensors are longitudinally attached along the dorsal side of the fingers, allowing for maximal detection of the axial strain variations induced by joint flexion and extension.

**Figure 2:**
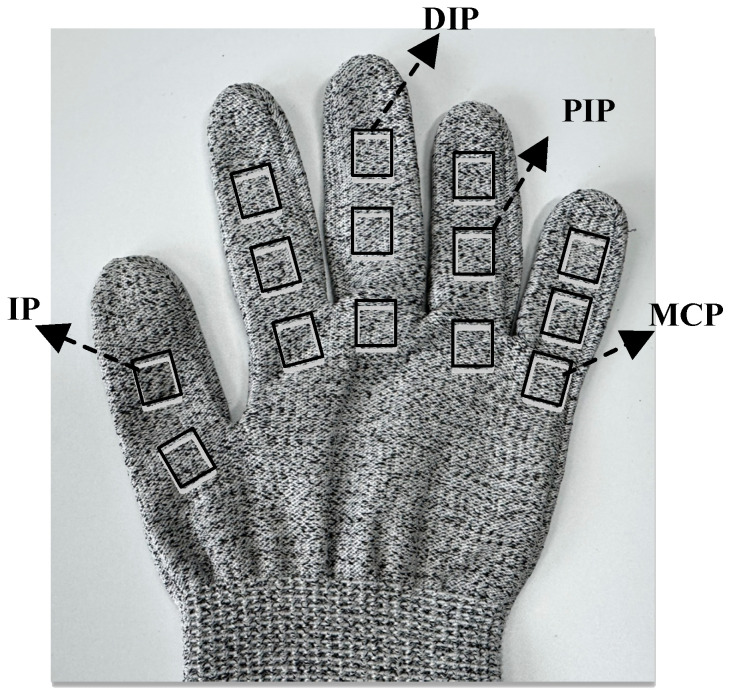
Schematic diagram of strain-gauge sensor layout.

Figure 2 illustrates the placement scheme of the strain sensors, showing placements across the primary flexion joints of the five fingers.

### 3.2. Experimental Setup and Data Acquisition Protocol

To evaluate the adaptability and stability of the strain signal acquisition system designed in this study under multi-user dynamic conditions, data collection experiments were conducted involving 10 healthy volunteers, including 7 males and 3 females, with the volunteers aged between 20 and 30 years. All participants had no neurological disorders or motor impairments. The entire experiment was carried out in a controlled laboratory environment, and informed consent was obtained from all participants.

Before data collection, a baseline calibration procedure was performed for each participant to minimize individual differences in signal baseline levels. Specifically, the reference voltage of each strain sensor was adjusted to 2.5 V under a relaxed, non-deformed hand posture, ensuring consistent signal initialization across subjects.

**Figure 3:**
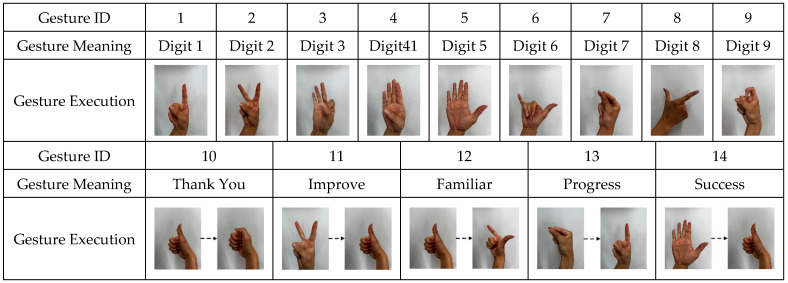
Classification and execution diagram for the dynamic gestures. Each gesture involves a continuous transition from an initial neutral state to the final hand posture. For gestures with intermediate transitional postures, arrows are used to indicate the direction of change between steps.

Since this study focuses on continuous and natural dynamic-gesture recognition, static gestures—which are mostly single-frame classifications with simple temporal structures—are not discussed further. As shown in Figure 3, the experiment was designed with 14 representative types of dynamic hand gestures. For each gesture, data were collected for 80 s, during which the participants continuously repeated the same gesture. The specific execution procedure was as follows: starting from an initial neutral state, the participant made a fist for 1 s, then completed a full hand gesture within 5 s, and finally returned to a resting state for 2 s before beginning the next cycle. Each dynamic gesture was performed in a cyclical manner, with each participant repeating the gesture 10 times, and each cycle lasting 8 s, resulting in 80 s of continuous signal recording per gesture. To ensure consistency and standardized timing, the entire data acquisition process was assisted by a screen-guided interface and voice prompt system, which provided synchronized instructions to guide participants through each stage of the motion.

## 4. Proposed Recognition Network

To address the challenges of dynamic-gesture recognition in multi-channel strain signals—such as insufficient local detail extraction, weak temporal modeling, and limited feature fusion capability—this paper proposes a lightweight neural network architecture named MACLiteNet, which integrates local–global feature fusion. The network is designed to collaboratively model local spatiotemporal features and global temporal dependencies while maintaining low computational complexity, thereby improving both the accuracy and robustness of dynamic-gesture recognition.

### 4.1. Overall Network Architecture

In dynamic-gesture recognition tasks based on multi-channel strain signals, a key challenge lies in simultaneously modeling both short-term local dynamics and long-term cross-channel dependencies to improve recognition accuracy and robustness. CNNs are effective in extracting local neighborhood features but are inherently limited by fixed receptive fields, making them less capable of capturing long-range dependencies. In contrast, Transformer architectures based on self-attention mechanisms have achieved significant success in global feature modeling in recent years [27,28]. Leveraging the complementary strengths of CNNs and Transformers in local and global modeling, this paper proposes a lightweight multi-branch hybrid attention network, named MACLiteNet. Through a module-level fusion strategy, the network jointly models the spatiotemporal characteristics of dynamic gestures, effectively enhancing action prediction performance based on strain signals, while maintaining low computational complexity.

**Figure 4:**
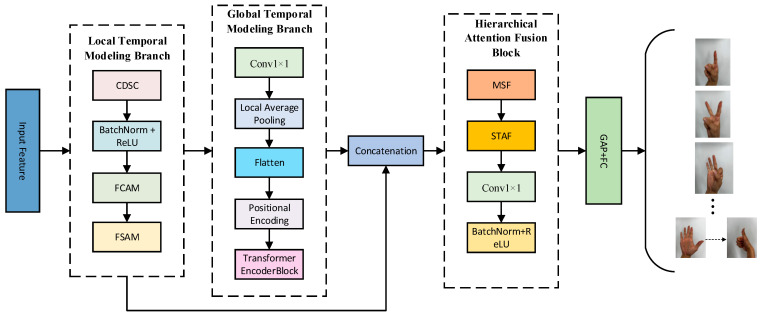
Overall architecture of the proposed lightweight multi-branch hybrid attention network, MACLiteNet.

As illustrated in Figure 4, the architecture of MACLiteNet consists of two feature-extraction branches and one fusion module:

- Local Temporal Modeling Branch (LTMB): Responsible for capturing short-term temporal dynamics and local features;
- Global Temporal Modeling Branch (GTMB): Designed to model long-range dependencies across time steps;
- Hierarchical Attention Fusion Block (HAFB): Integrates local and global features while enhancing channel selectivity.

The entire network takes multi-channel strain signal sequences as input. After preprocessing, the data are sequentially fed into LTMB and GTMB to extract feature representations at different temporal scales. These features are then fused and enhanced within the HAFB; this is followed by a final classifier to produce the gesture prediction output.

Table 2 summarizes the detailed configuration of each module within MACLiteNet, including the corresponding layer types, output sizes, and functional descriptions.

### 4.2. Local Temporal Modeling Branch

In sequential signal modeling tasks, the fine-grained extraction of local dynamic features is one of the key factors for achieving high-accuracy dynamic-gesture recognition. Unlike image data, strain signals exhibit strong temporal causality and multi-channel input characteristics, which impose stricter requirements on the model—necessitating not only strong temporal perception but also effective channel selectivity and computational efficiency.

To meet these demands, this study proposes the LTMB module, which constructs a feature-extraction pathway with inherent temporal awareness and effectively enhances the model’s ability to capture short-term dynamic variations. The LTMB consists of two stages: the front-end backbone employs a causal convolutional structure to extract temporal features, while the back-end introduces an attention mechanism to enhance the model’s responsiveness to both critical channels and key frames.

In the feature-extraction stage, the LTMB adopts a Causal Depthwise Separable Convolution (CDSC) module, inspired by the concept of causal convolution introduced in Temporal Convolutional Networks (TCN) [29]. This module applies unidirectional padding along the temporal dimension, preserving only current and past time steps to ensure unidirectional information flow, thereby preventing future-frame information from interfering with the current feature representation.

**Figure 5:**
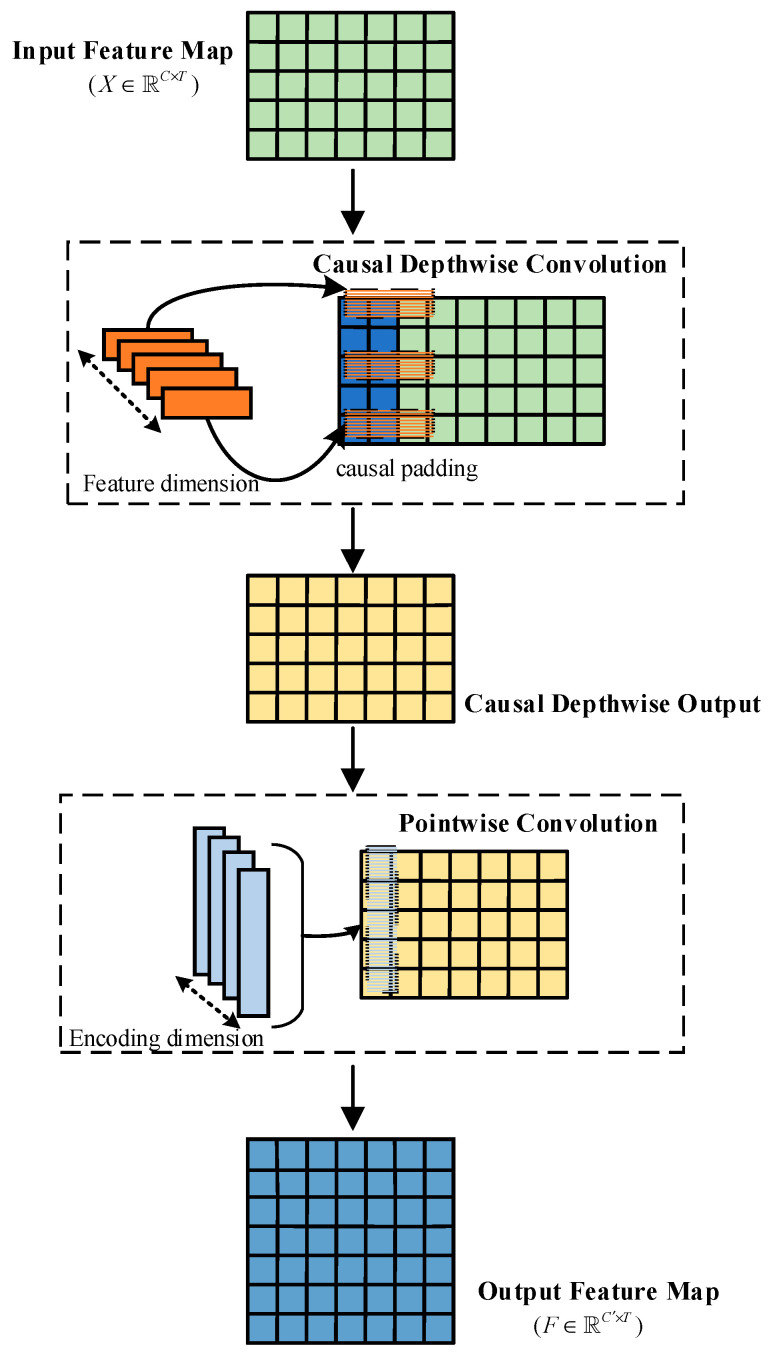
Structure of the Causal Depthwise Separable Convolution block.

The CDSC module consists of two subcomponents: causal depthwise convolution, which extracts temporal features independently within each channel, and pointwise convolution, which integrates inter-channel dependencies. As shown in Figure 5, let the input feature be denoted as $X\in {\mathbb{R}}^{C\times T}$, where C is the number of sensor channels and T is the temporal length. First, a one-dimensional causal convolution is applied independently to each channel to extract its local dynamic features. The computation is defined as

(1) $$F_{c}(t)=\overset{K-1}{\underset{k=0}{\sum }}w_{c}(k)\cdot X_{c}(t-k)$$

where $w_{c}(k)$ denotes the convolution kernel parameter at position k along the temporal axis for channel c, and $X_{c}(t-k)$ represents the input feature of channel c at time step t−k.

Subsequently, to model inter-channel dependencies, the CDSC applies a pointwise convolution at each time step to fuse features across all channels, generating more discriminative representations. The computation is defined as

(2) $$F_{depth}(t)={\left[F_{1}(t),F_{2}(t),\ldots ,F_{C}(t)\right]}^{T}$$

(3) $$F_{t}=W\cdot F_{depth}(t)$$

where $F_{depth}(t)\in {\mathbb{R}}^{C}$ denotes the output feature of the depthwise convolution at time step t, $W\in {\mathbb{R}}^{C^{\prime }\times C}$ is the weight matrix of the pointwise convolution, and $C^{\prime }$ is the number of output channels. The final output feature is represented as $F\in {\mathbb{R}}^{C^{\prime }\times T}$, which is subsequently fed into the attention module.

In the feature enhancement stage, the LTMB integrates a Feature Channel Attention Module (FCAM) and a Feature Spatial Attention Module (FSAM) in a cascaded configuration [30], aiming to enhance the model’s sensitivity to both critical channels and key temporal regions. Unlike the commonly used parallel design, this work adopts a serial attention strategy: FCAM is first applied to extract salient channel responses, followed by FSAM to focus on important time frames, thereby modeling attention progressively from the channel dimension to the temporal dimension.

This design improves feature selectivity and discriminative capability without altering the original structures of the attention modules. It is worth noting that, to retain the attention mechanism’s strength in global feature modeling, no causality constraint is imposed on FCAM and FSAM, allowing them to fully utilize information across the entire temporal sequence.

### 4.3. Global Temporal Modeling Branch

After extracting local spatiotemporal features through the LTMB branch, the GTMB is introduced to further capture long-range dependencies across time steps. This branch leverages a combination of linear projection and a temporal modeling module to effectively learn contextual patterns within complex gesture sequences.

First, the output feature $X\in {\mathbb{R}}^{C\times T\times W}$ from the LTMB is processed through channel compression using a 1 × 1 convolution for linear projection, mapping the original channel dimension C to a new dimension $C^{\prime }$. Subsequently, average pooling is applied on the width dimension W to reduce spatial redundancy and improve computational efficiency. The processed features are flattened into a time series tensor of length T:

(4) $$Z={\left[z_{1},z_{2},\ldots ,z_{T}\right]}^{T}\in {\mathbb{R}}^{T\times C^{\prime }}$$

where $z_{t}\in {\mathbb{R}}^{C^{\prime }}$ denotes the feature representation at time step t.

To enhance the model’s awareness of temporal order, a positional encoding matrix $P\in {\mathbb{R}}^{T\times C^{\prime }}$ is added to the sequence features, forming the input to the Transformer encoder:

(5) $$H^{\left(0\right)}=Z+P$$

where $H^{\left(0\right)}$ is the initial input embedding that incorporates both feature content and temporal positional information.

Next, a stack of Transformer encoder layers is introduced to model global contextual relationships. Each encoder layer consists of two sub-modules: Multi-Head Attention (MHA) and a Feedforward Neural Network (Multi-Layer Perceptron, MLP). To enhance training stability and information flow, residual connections and Layer Normalization are applied before and after each sub-module. Let the input to the l-th encoder layer be $H^{\left(l-1\right)}$; the computation process of this layer is as follows:

(6) $$U^{\left(l\right)}=MHA\left(LN\left(H^{\left(l-1\right)}\right)\right)+H^{\left(l-1\right)}$$

(7) $$H^{\left(\;l\;\right)}=MLP\left(LN\left(U^{\left(\;l\;\right)}\right)\right)+U^{\left(\;l\;\right)}$$

where $U^{\left(l\right)}$ denotes the intermediate representation after the self-attention operation, and $H^{\left(\;l\;\right)}$ is the output of the l-th encoder layer.

The MHA module applies a multi-head scaled dot-product attention mechanism across all time steps to capture temporal dependencies at multiple scales. The MLP consists of two fully connected layers with a ReLU activation function in-between, enhancing the network’s capacity for nonlinear representation. By stacking multiple Transformer encoder layers, the GTMB can effectively model long-range contextual relationships across time steps, compensating for the limitations of the local modeling branch in capturing extended dependencies. This design significantly enhances the overall network’s ability to model the evolution of complex dynamic gestures.

### 4.4. Hierarchical Attention Fusion Block

To fully integrate the multi-scale dynamic-gesture features extracted from the local and global branches, this study proposes the HAFB module. The module consists of two subcomponents: a Multi-Scale Feature Enhancement (MSF) module, designed to enrich spatial–temporal contextual information, and a Dual-Pooling Channel Attention (DPCA) module, aimed at improving discriminability along the channel dimension. These two components work in tandem to enhance both the diversity of temporal–spatial representations and the selectivity of channel-wise responses, enabling more robust feature fusion across branches.

#### 4.4.1. Multi-Scale Feature Enhancement

**Figure 6:**
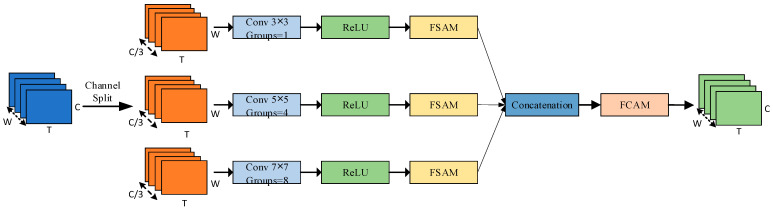
Structure of the MSF module.

The MSF module is designed to enrich spatial–temporal representations by integrating multi-branch convolutional features with hierarchical attention. As illustrated in Figure 6, the MSF employs three parallel convolutional branches, each configured with different kernel sizes $k_{i}\in \left\{3,5,7\right\}$ and group settings $g_{i}\in \left\{1,4,8\right\}$, to capture contextual information at varying receptive fields. The input features are first channel-wise average pooled along the temporal dimension and then divided into three groups, which are processed by the corresponding branches with ReLU activation. To enhance discriminability at each scale, the output of each branch is refined using FSAM, and the multi-scale features are concatenated along the channel dimension. The fused representation is then further recalibrated by FCAM to strengthen inter-channel dependencies. Ultimately, the enhanced feature map output produced by the MSF possesses both multi-scale contextual information and cross channel attention perception capabilities, providing a more semantic expression for subsequent fusion discrimination.

Let the enhanced feature be denoted as $X_{\mathrm{fused}}\in {\mathbb{R}}^{C\times T\times W}$ where C is the number of concatenated channels, T the temporal length, and W the spatial width. The process can be formulated as

(8) $$X_{\mathrm{fused}}=\left[X_{1},X_{2},X_{3}\right],\;X_{i}\in {\mathbb{R}}^{\frac{C}{3}\times T\times W}$$

(9) $$F_{i}=\mathrm{ReLU}\left({\mathrm{Conv}}_{k_{i},g_{i}}\left(X_{i}\right)\right),\;i=1,2,3$$

(10) $$Z_{i}=\mathrm{FSAM}\left(F_{i}\right),\;i=1,2,3$$

(11) $$Z=\left[Z_{1}:Z_{2}:Z_{3}\right]$$

(12) $$X_{MSF}=FCAM\left(Z\right)$$

where ${\mathrm{Conv}}_{k_{i},g_{i}}(\cdot )$ denotes a grouped convolution operation with kernel size $k_{i}$ and group number $g_{i}$, and ReLU represents the activation function. FSAM and FCAM denote the spatial and channel attention modules, respectively.

#### 4.4.2. Dual-Pooling Channel Attention

Although the MSF module has already achieved multi-scale spatial–temporal fusion, the resulting features may still exhibit uneven channel-wise responses, leaving redundancy or missing cues. To further enhance the model’s ability to discriminate between channels and preserve the structural integrity of feature representations, we designed a DPCA module as a fine-grained channel attention component.

**Figure 7:**
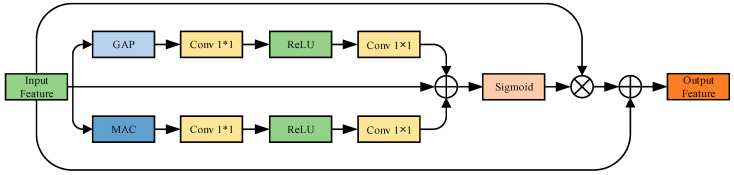
Structure of the DPCA module.

As shown in Figure 7, DPCA adopts a dual-path attention design inspired by the “squeeze–excitation” concept in the SE module and the dual-path strategy in CBAM [31]. Let the fused feature map output from MSF be denoted as $F\in R^{C\times T\times W}$, where C is the number of channels, T is the temporal length, and W is the spatial width. To further model the global dependencies in the channel dimension, the DPCA module performs AvgPool and MaxPool operations on the T and W dimensions, respectively, to aggregate the two-dimensional spatial regions (T, W) corresponding to each channel in the channel dimension and extract the channel’s global statistical features. Two pooling methods model the response strength of channels from different perspectives, reflecting the overall activity and extreme response ability of the channels, respectively, which helps enhance the network’s perception ability relative to key channels.

Subsequently, the two pooled channel descriptors are fed into a shared two-layer convolutional neural network structure, which consists of two 1 × 1 convolutional layers and a ReLU activation function. The results of average pooling and max pooling are combined and passed through a sigmoid function to generate the final channel attention weights $M_{c}\left(I\right)\in R^{C\times 1\times 1}$, which are used to reweight the original feature map F.

The attention weights are then multiplied with F in a channel-wise manner to obtain the enhanced feature map $M_{c}\left(F\right)$. To avoid the loss of information due to attention modulation while preserving the original structural responses, a residual connection is introduced. The final output of DPCA is obtained by element-wise summation of the input and the reweighted features:

(13) $$I_{avg}^{c}=AvgPool\left(F\right)$$

(14) $$I_{\max}^{c}=MaxPool\left(F\right)$$

(15) $$\begin{array}{c} M_{c}\left(I\right)\;=\sigma [f_{2}^{1\times 1}(r(f_{1}^{1\times 1}(I_{avg}^{c})))]\\ \;\;\;\;+\sigma [f_{2}^{1\times 1}(r(f_{1}^{1\times 1}(I_{\max}^{c})))] \end{array}$$

(16) $$M_{c}\left(F\right)=M_{c}\left(I\right)\times F$$

(17) $$Y=M^{c}\left(F\right)+F$$

where $f_{1}^{1\times 1}$ denotes the first 1 × 1 convolution layer, with an input channel size of d and an output channel size of d/16; $f_{2}^{1\times 1}$ denotes the second 1 × 1 convolution layer, with an input size of d/16 and output size of d; σ denotes the sigmoid function; and r denotes the ReLU activation function.

In summary, as an integrated fusion unit, the HAFB module combines the MSF and DPCA to effectively integrate the heterogeneous features extracted from the LTMB and GTMB branches. This enhances the discriminative power and semantic completeness of the fused representation, making it a key contributor to the overall performance improvement of the network.

## 5. Experimental Settings and Data Resources

This section introduces the datasets, preprocessing strategies, training configurations, and evaluation protocols adopted for model performance assessment. The experiments are conducted using two sources of data. The first is the self-constructed strain-gauge dataset developed in this study, which is used to evaluate the model’s effectiveness in recognizing gestures from real-world strain signals. The second involves the publicly available sEMG dataset NinaPro DB1—Exercise 2, which is employed to assess the proposed model’s transferability in cross-modal gesture-recognition scenarios.

### 5.1. Strain-Gauge Dataset Partitioning and Processing

The self-constructed dataset in this study comprises dynamic-gesture recordings from 10 participants, covering 14 gesture classes. The data collection procedure is described in detail in Section 3.2. Raw signals are stored in CSV format, and each file corresponds to one complete gesture cycle and contains timestamps, voltage readings from 14 strain channels, and gesture labels. The sampling rate is set to 100 Hz.

To extract temporally discriminative features suitable for model learning, a fixed sliding window strategy is applied. Each window has a length of 400 samples with a stride of 200. Subsequently, the data are downsampled by a factor of 2 along the temporal dimension, yielding a two-dimensional feature matrix of size [14 × 200]. For consistency and compatibility with classification models, all gesture labels are re-encoded into integer indices starting from 1.

### 5.2. Public sEMG Dataset: NinaPro DB1

To evaluate the model’s adaptability and transferability in the domain of sEMG, this study utilizes data from five subjects in the DB1—Exercise 2 subset of the NinaPro database. The dataset was collected by the IDSIA research institute and contains forearm sEMG signals from 27 healthy subjects [32,33].

In Exercise 2, participants are instructed to perform 17 types of dynamic gestures, such as finger flexion/extension, fist clenching, and wrist rotations. Each gesture is repeated six times, with each repetition lasting approximately 5 s, followed by a 3 s rest interval between actions [34]. Due to its standardized protocol, rich gesture diversity, and high-quality annotations, this dataset has become a widely adopted benchmark for evaluating sEMG-based gesture-recognition methods.

### 5.3. Network Training and Validation Configuration

During training, the Adam optimizer is employed, a feature which integrates momentum and adaptive learning rate mechanisms, making it well-suited for non-convex optimization problems. The loss function is set to cross-entropy loss, which is commonly used in multi-class classification tasks.

The initial learning rate is set to 0.001, with a batch size of 32. A cosine annealing learning rate scheduler is adopted to dynamically adjust the learning rate during training. To enhance the model’s generalization capability, L2 regularization is applied to all weight parameters, along with a coefficient of 0.001. The total number of training epochs is set to 50. The selection of these key hyperparameters was guided by Bayesian optimization, aiming to ensure the robustness of the model’s performance. A detailed comparative analysis of this process is not presented in this paper.

To ensure a fair evaluation and avoid potential overfitting or data leakage, a sample-level five-fold cross-validation strategy based on cycle-level grouping was adopted. Specifically, all sliding windows generated from the same 8 s gesture cycle were treated as a group during dataset splitting, ensuring that no window segments from the same gesture cycle appeared simultaneously in both the training and testing sets. This approach comprehensively evaluates the model’s generalization ability under known-user scenarios while effectively eliminating the risk of data leakage.

### 5.4. Evaluation Metrics

To comprehensively evaluate the performance of the proposed MACLiteNet framework in the gesture classification task, multiple metrics are employed, including accuracy, recall, precision, and *F*1-score. These metrics provide a holistic view of the model’s classification effectiveness from different perspectives. The specific formulations are as follows:

(18) $$\mathrm{Accuracy}=\frac{\mathrm{TP}+\mathrm{TN}}{\mathrm{TP}+\mathrm{FN}+\mathrm{FP}+\mathrm{TN}}$$

(19) $$\mathrm{Precision}=\frac{\mathrm{TP}}{\mathrm{TP}+\mathrm{FP}}$$

(20) $$\mathrm{Recall}=\frac{\mathrm{TP}}{\mathrm{TP}+\mathrm{FN}}$$

(21) $$F1=2\times \frac{\mathrm{Precision}\times \mathrm{Recall}}{\mathrm{Precision}+\mathrm{Recall}}$$

where TP denotes the number of samples predicted to be Class x and actually belonging to Class x, FP denotes the number of samples predicted to be Class x but actually belonging to other classes, FN denotes the number of samples that truly belong to Class x but are misclassified into other classes, and TN denotes the number of samples that are neither predicted to be Class x nor actually belonging to Class x.

## 6. Experimental Results and Analysis

To evaluate the effectiveness of the proposed MACLiteNet in gesture recognition, a series of qualitative and quantitative experiments were conducted on the self-constructed strain dataset. Additionally, the NinaPro DB1 dataset was used to assess its cross-modal generalization. Ablation studies were conducted to assess the contributions of individual modules, while cross-modal evaluations and comparisons with mainstream models were performed to verify the generalization ability and overall performance advantages of MACLiteNet in terms of accuracy and efficiency.

### 6.1. Ablation Study

To comprehensively evaluate the contribution of each proposed module to the overall gesture-recognition performance, systematic ablation experiments were conducted, focusing on the effectiveness of the LTMB, MSF, and DPAC modules. Based on these components, several network configurations were constructed for comparison, including Baseline, Baseline + LTMB, Baseline + MSF, Baseline + DPAC, Baseline + MSF + DPAC, and the full Proposed model.

The baseline network served as a control group, in which all attention and fusion modules were removed, retaining only the basic depthwise separable convolution structure. Specifically, the causal depthwise separable convolution in the original LTMB was replaced with standard depthwise separable convolution; the MSF module was bypassed using an identity-mapping-without-multi-scale enhancement; and in the fusion stage, only a 1 × 1 convolution followed by ReLU activation was used for channel integration, omitting the residual attention mechanism in the DPAC module.

Based on the four evaluation metrics introduced in Section 5.4—accuracy, recall, precision, and *F*1-score—Table 3 summarizes the ablation results under different network configurations.

As shown in Table 3, incorporating the LTMB module led to performance improvements of 3.22% in accuracy and recall, 3.21% in precision, and 3.23% in *F*1-score, demonstrating the positive effects of causal convolution and the dual-branch attention mechanism in temporal sequence modeling.

Compared to the LTMB, the MSF module produced more significant performance gains. The accuracy, recall, and *F*1-score of the MSF all reached 98.79%, while the precision rose to 98.80%. This indicates that the multi-scale feature fusion mechanism is more advantageous in modeling complex gesture patterns.

However, when the DPAC module was introduced in isolation, all performance metrics declined. This can be attributed to the fact that the DPAC was originally designed to operate in conjunction with multi-scale features. Without the MSF module, the eDPAC failed to extract discriminative information, causing the attention mechanism to degenerate into global averaging, or even learn ineffective weights. This suggests that the DPAC module struggles to capture inter-channel importance distributions in the absence of multi-source inputs, reducing the fusion process to near-linear compression.

Further experiments showed that jointly introducing both the MSF and DPAC modules into the baseline network yields significant improvements across all evaluation metrics (see Table 3), outperforming configurations that use either module individually. Although the MSF alone already provides considerable performance gains, the presence of the DPAC enables a more effective weighting and integration of multi-scale features, further enhancing the model’s overall discriminative power.

Finally, by integrating all three modules—the LTMB, MSF, and DPAC—into the baseline network, the proposed full architecture achieved the highest performance, with an accuracy of 99.71% across all four metrics. These results confirm the synergistic effect of the proposed modules and demonstrate that MACLiteNet achieves state-of-the-art recognition performance for multi-channel strain-based gesture classification.

### 6.2. Performance Visualization

**Figure 8:**
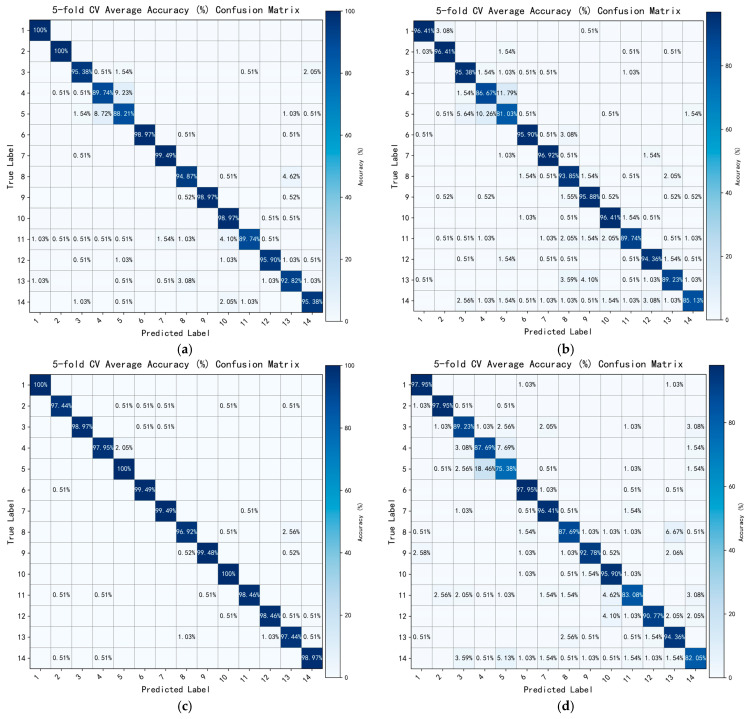

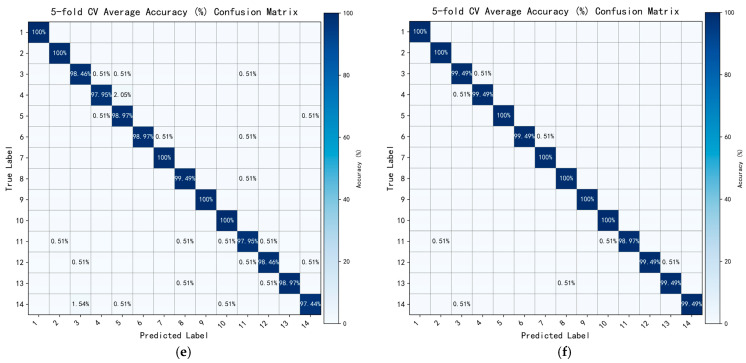
Average confusion matrices over five-fold cross-validation under different network configurations: (**a**) Baseline; (**b**) Baseline + LTMB; (**c**) Baseline + MSF; (**d**) Baseline + DPAC; (**e**) Baseline + MSF + DPAC; and (**f**) Proposed.

To intuitively illustrate the classification performance of the dynamic-gesture recognition network under different module configurations, confusion matrices corresponding to each configuration are plotted, as shown in Figure 8. The figure presents the average classification performance across 14 gesture classes under five-fold cross-validation. The horizontal axis represents the predicted classes, while the vertical axis denotes the ground truth labels.

Inspired by the quantitative evaluations in the previous ablation study, this section adopts a module-wise visualization approach to analyze how different architectural components affect the classification outcomes.

As shown in Figure 8a, the baseline network, lacking any enhancement modules, exhibits relatively low overall accuracy. Significant confusion is observed among several similar gesture classes, particularly between Class 5 and Class 14, in which performance is unstable and the recognition rate for certain classes drops below 85%. This indicates that, without contextual modeling or feature enhancement, the network struggles to distinguish complex or rhythmically similar gestures.

When the channel attention module LTMB is added to the baseline (Figure 8b), the overall discriminative capability of the model is noticeably improved. Several classes achieve recognition rates above 95%, especially those with subtle differences in channel response distributions. This confirms the positive effect of channel attention mechanisms in enhancing discriminative feature representations.

Figure 8c presents the classification results after introducing the multi-scale MSF branch structure. This configuration significantly improves recognition performance across all gesture classes, particularly for previously ambiguous gestures (e.g., Classes 11 to 14), the accuracy of which sees a substantial increase. These results suggest that the multi-scale receptive fields provided by the MSF help the network better adapt to variations in gesture duration, amplitude, and rhythm, thereby enhancing generalization.

However, when only the DPAC module is incorporated (Figure 8d), the recognition performance not only fails to improve, but also degrades in several classes. For example, gestures in Classes 3, 5, and 8—those with highly similar activation patterns—experience increased confusion. This suggests that in the absence of multi-source inputs (such as those provided by the MSF), the DPAC module’s channel reconstruction capability deteriorates, leading to ineffective feature fusion and even disruptive interference in feature distribution.

When the MSF and DPAC are used in combination (Figure 8e), the network demonstrates outstanding performance across all classes. Most gesture classes exceed 98% in recognition accuracy, including previously challenging gestures like Classes 12 and 13, which achieve 97.95% and 98.46%, respectively. This result highlights the complementary strengths of the MSF and DPAC, in which multi-scale feature representation and attention-based channel reconstruction jointly enhance the model’s ability to capture fine-grained differences.

Finally, as shown in Figure 8f, the proposed complete model achieves the best performance across all gesture classes, with an overall accuracy of 99.71%. Only Class 11 falls slightly short, yet it still reaches 98.97%. These results further confirm the superiority and high generalization capability of the proposed network in dynamic-gesture recognition, particularly in distinguishing complex, rhythmically similar, or structurally ambiguous gestures.

### 6.3. Cross-Modal Generalization and Transfer Evaluation

To assess the adaptability and generalization capability of the proposed dynamic-gesture recognition model across different sensing modalities, we further evaluate its cross-modal performance by transferring the model trained on the self-constructed strain-gauge dataset to the publicly available sEMG dataset, NinaPro DB1—Exercise 2. This experiment aims to determine whether the model is capable of recognizing gestures across heterogeneous modalities, thereby validating its architectural generality and transferability.

Considering the inherent differences in signal characteristics and temporal structures between the strain sensor and sEMG modalities, modality-specific input preprocessing was performed. Specifically, for the strain data, a sliding window of 400 time steps was applied, followed by a uniform downsampling operation with a factor of two, resulting in a final input sequence length of 200 time steps. For the sEMG data, given its distinct temporal structure and gesture activation patterns, a longer sliding window of 800 time steps was utilized to ensure sufficient coverage of relevant signal dynamics. The same downsampling operation was performed, yielding a final input sequence length of 400 time steps. The original amplitude information of both signal types was preserved without additional normalization or filtering. To adapt to the different input shapes, only the first convolutional layer of MACLiteNet was modified accordingly for each modality, while the overall network architecture, data segmentation strategy, and training configurations remained unchanged.

**Figure 9:**
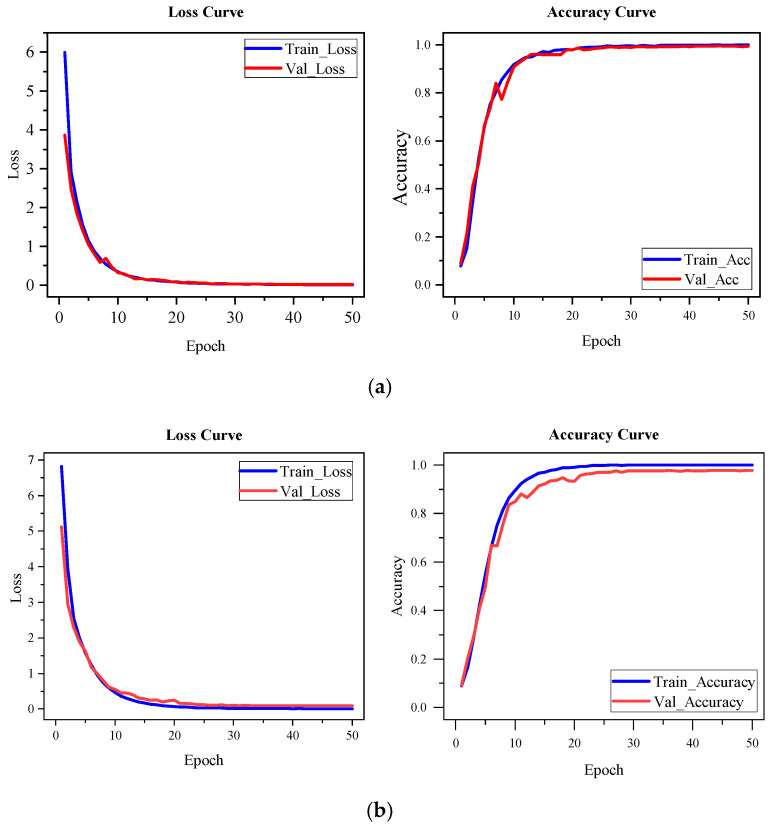
Training process comparison for the proposed model, on different modalities: (**a**) training accuracy and loss curves on the self-constructed strain-gauge dataset; (**b**) training accuracy and loss curves on the public sEMG dataset (NinaPro DB1).

To provide a comprehensive comparison of the model’s training behavior and performance across both modalities, Figure 9 presents the training accuracy and loss curves on the self-constructed strain-gauge dataset and the NinaPro DB1 dataset, respectively. As shown in Figure 9a, when trained on the strain dataset, the model gradually improves from random initialization, with a stable convergence process and high final accuracy. This demonstrates the effectiveness of the proposed architecture in modeling temporal patterns from strain signals.

Figure 9b illustrates the training curves after transferring the model to the sEMG dataset. It can be observed that the model achieves high accuracy from the early stages of training and converges rapidly within a few epochs. The training process is smooth, with no significant fluctuations. These results indicate that the temporal structure modeling and inter-channel dependency representations learned from the strain modality remain effective and generalizable in the sEMG domain. The final test accuracy stabilizes at 98.45%, further confirming the cross-modal applicability of the proposed network in dynamic-gesture recognition.

### 6.4. Comparison with Existing Methods

To comprehensively evaluate the classification performance of the proposed dynamic-gesture recognition model on multi-channel strain-gauge data, four representative deep learning models were selected as baseline methods: the traditional convolutional neural network VGG16 [35]; the Transformer-based architecture ViT [20]; the lightweight hybrid model MobileViT [22], which combines convolutional and Transformer components; and the widely adopted CNN-LSTM model [36]

All models were trained and tested on the constructed multi-channel strain-based gesture-recognition dataset, using a unified data preprocessing pipeline and identical input shapes and training strategies to ensure fair comparisons.

**Figure 10:**
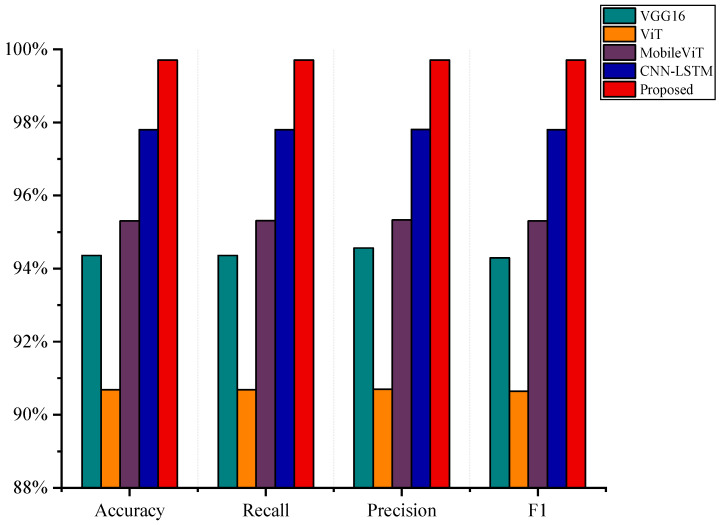
Performance comparisons of different models relative to key classification metrics.

Table 4 summarizes the performance of all models in terms of key evaluation metrics, including accuracy, recall, precision, *F*1-score, number of parameters, and computational complexity (FLOPs). To provide a more intuitive comparison of classification effectiveness, Figure 10 further presents a bar chart illustrating four core metrics: Accuracy, Macro-*F*1, Weighted-*F*1, and Precision. As observed from the results, the proposed model consistently outperforms all baseline methods across all evaluation metrics. Compared to VGG16, ViT, MobileViT, and the widely adopted CNN-LSTM model, it achieves superior overall performance, demonstrating its ability to effectively extract both spatial and temporal features while maintaining high classification accuracy and computational efficiency within a lightweight design.

It is worth noting that the proposed MACLiteNet contains only 0.22 million parameters and requires 0.10 GFLOPs, based on theoretical calculations using standard floating-point operations, highlighting its lightweight characteristics. Although real-time gesture recognition has not yet been implemented directly on embedded hardware in this study, the compact architecture and low computational complexity indicate its strong potential for deployment on resource-constrained platforms, such as wearable devices or edge processors. Future work will explore real-time implementation and evaluation on embedded hardware.

Specifically, the proposed model achieves 99.71% in Accuracy, Macro-*F*1, and Weighted-*F*1, and 99.72% in Precision. Compared to VGG16, these results represent improvements of 5.35%, 5.42%, 5.15%, and 5.43%, respectively. Compared to MobileViT, the proposed model shows gains of 4.41%, 4.41%, 4.41%, and 4.45% in the same metrics. In addition, the model contains approximately only 0.22 million parameters and requires 0.10 GFLOPs, which are significantly lower than those of VGG16 (84.9M parameters and 0.35 GFLOPs). Furthermore, compared to the widely adopted CNN-LSTM model, which achieves 97.80% in all core metrics, the proposed architecture still shows noticeable gains of approximately 1.91%, demonstrating its superior capability in joint spatial–temporal feature extraction for dynamic-gesture recognition.

In addition to its excellent performance, the proposed MACLiteNet maintains remarkable structural compactness and computational efficiency. It contains approximately only 0.22 million parameters and requires 0.10 GFLOPs, which are significantly lower requirements than the 84.9 million parameters and 0.35 GFLOPs of VGG16, and also lower than those of the CNN-LSTM model, which has 0.41 million parameters. Although the FLOPs of the CNN-LSTM are slightly lower, its overall classification performance is inferior to that of the proposed model, indicating that MACLiteNet achieves a better trade-off between accuracy and model complexity. Compared to MobileViT, MACLiteNet requires moderately higher computational resources, but delivers substantial performance improvements.

These results demonstrate that the proposed MACLiteNet not only delivers state-of-the-art classification performance but also achieves an excellent balance between accuracy, structural compactness, and computational efficiency.

### 6.5. Limitations and Practical Considerations

While the proposed MACLiteNet demonstrates excellent performance under controlled experimental conditions, several challenges may arise in real-world deployment scenarios. In practice, strain sensor signals are susceptible to various sources of noise, including electromagnetic interference, skin movement artifacts, and improper sensor placement. These factors may degrade signal quality and stability, subsequently affecting recognition accuracy.

Although the integration of attention mechanisms and multi-scale feature fusion is intended to enhance model robustness, the system may still exhibit sensitivity to sensor placement variability and individual physiological differences. Additionally, despite efforts to prevent overfitting, the possibility of model overfitting relative to specific sensor configurations or controlled laboratory environments cannot be entirely excluded, especially given the high recognition accuracy achieved.

Future work will focus on extensive testing under conditions that are more realistic and dynamic, including the use of different sensor placements, user-independent evaluations, and long-term continuous use. Further improvements to sensor materials and attachment mechanisms will also be explored to reduce signal drift and enhance system stability in practical applications.

## 7. Conclusions

To address the challenge of accurate and efficient dynamic-gesture recognition, this study proposes a lightweight hybrid attention network, MACLiteNet, based on multi-channel strain signals acquired via a flexible and skin-conformal strain-gauge sensing system. The model is designed to achieve precise multi-scale dynamic-gesture modeling and classification. Comprehensive qualitative and quantitative evaluations utilizing the constructed multi-channel strain-based gesture dataset demonstrate the superiority of the proposed method in terms of recognition accuracy, model efficiency, and generalization capability. The main contributions of this work are summarized as follows:

1. A standardized multi-channel strain signal acquisition and dataset construction scheme was developed, incorporating joint-level sensor layout and a unified gesture acquisition protocol, resulting in a structured and reproducible dynamic-gesture dataset that supports high-quality model training and evaluation.
2. A Local Temporal Modeling Branch (LTMB) was proposed that integrates causal convolution and attention mechanisms at both the channel and frame levels to capture fine-grained local temporal variations and enhance key dynamic features.
3. A Multi-Scale Feature Enhancement (MSF) module was designed, with the aim of extracting contextual information across diverse receptive fields using multi-branch convolutions and spatial attention, thereby improving the network’s ability to represent complex gesture structures.
4. A Dual-Pooling Channel Attention (DPCA) module was constructed, combining global pooling and residual fusion to perform refined channel-wise feature recalibration, enhancing both model discriminability and generalization.

In future work, we plan to further explore the deployment adaptability of the proposed model in real-world scenarios, extend its cross-modal interaction capabilities, and promote its application in human–computer interaction, rehabilitation training, and wearable intelligent systems.

## Figures and Tables

**Table 1:** Main parameters of the configuration of the system.

Case	Parameter	Value
1	Sampling frequency	100 Hz
2	ADC channels	5 parallel ADC ports (ESP32 internal)
3	Number of signal channels	14 (via MUX multiplexing)
4	Signal conditioning	Wheatstone bridge + two-stage amplifier
5	Wireless transmission	Wi-Fi (ESP32 built-in)
6	Supply voltage	3.3 V
7	Reference voltage	2.5 V (baseline)
8	PCB size	3.6 cm × 3.6 cm
9	Processor	Xtensa^®^ dual-core 32-bit LX6 (ESP32 SoC)
10	Data rate (to PC)	~100 samples/second × 14 channels

**Table 2:** Detailed module configuration of the proposed MACLiteNet.

Module	Layer Type and Parameters	Output Size	Description
Input	-	(32, 1, 14, 200)	Multi-channel strain signal input
CDSC	Depthwise Separable Conv with causal padding	(32, 128, 200, 200)	Local dynamic feature extraction with causal constraint
LTMB	CDSC → FCAM → FSAM	(32, 128, 200, 200)	Enhances local channel and temporal feature representation
GTMB	1 × 1 Conv → Width-wise AvgPool (/4) → Positional Encoding → 2-layer Lightweight Transformer	(32, 64, 200, 50)	Models global temporal dependencies
Feature Concatenation	-	(32, 192, 200, 50)	Concatenation of local and global features
MSF	3 × 3, 5 × 5, 7 × 7 Multi-branch Conv → FSAM for each branch → Concatenation → FCAM	(32, 192, 200, 50)	Multi-scale feature fusion with spatial and channel enhancement
DPCA	AvgPool + MaxPool → Two 1 × 1 Convs → Sigmoid → Residual Connection	(32, 192, 200, 50)	Channel-wise feature enhancement and redundancy reduction
HAFB	MSF → DPCA → 1 × 1 Conv → BN → ReLU	(32, 192, 200, 50)	Hierarchical attention fusion for global feature integration
Classifier	1 × 1 Conv → BN → ReLU → GAP → Fully Connected Layer	(32, 14)	Generates classification probabilities for 14 gesture categories

**Table 3:** Ablation study results under different module configurations.

Method	Accuracy	Recall	Precision	*F*1
Baseline	92.38%	92.38%	92.44%	92.37%
Baseline + LTMB	95.60%	95.60%	95.65%	95.60%
Baseline + MSF	98.79%	98.79%	98.80%	98.79%
Baseline + DPCA	90.66%	90.66%	90.69%	90.60%
Baseline + MSF + DPCA	99.05%	99.05%	99.05%	99.05%
Proposed	99.71%	99.71%	99.71%	99.71%

**Table 4:** Comparative results for different models on dynamic-gesture recognition.

Method	Accuracy	Recall	Precision	*F*1	Parameters	FLOPs
VGG16	94.36%	94.36%	94.56%	94.29%	84.8946	0.3548
ViT	90.69%	90.69%	90.7%	90.645	1.1761	0.0901
MobileViT	95.30%	95.31%	95.33%	95.30%	0.0238	0.0009
CNN-LSTM	97.80%	97.80%	97.81%	97.80%	0.4143	0.0027
Proposed	99.71%	99.71%	99.71%	99.71%	0.2213	0.1

## Data Availability

The dataset used in this study was collected as part of an ongoing project within the research laboratory and is subject to confidentiality agreements. As such, the data cannot be shared publicly at this stage.
